# Assessment of the WHO non-communicable diseases kit for humanitarian emergencies in South Sudan: a retrospective, prospective, observational study

**DOI:** 10.1186/s13031-023-00525-w

**Published:** 2023-06-05

**Authors:** Ahmad Hecham Alani, Laura Miller, Bhavika Darji, Isaac Waweru, Aston Benjamin Atwiine, Marcello Tonelli, Joseph Lou Kenyi Mogga, Ali Adams, Lilian Ndinda, Said Jongo, Lilian Kiapi

**Affiliations:** 1 International Rescue Committee, London, UK; 2 Independent Researcher, London, UK; 3 International Rescue Committee, New York City, NY USA; 4 International Rescue Committee, Nairobi, Kenya; 5 International Rescue Committee, Kampala, Uganda; 6 Department of Medicine, University of Calgary, Calgary, AB Canada; 7 WHO Country Office, Ministry of Health, Ministerial Complex, Juba, South Sudan; 8 International Rescue Committee, Juba, South Sudan

**Keywords:** Noncommunicable diseases, Emergency kits, Humanitarian settings, Conflict, South Sudan

## Abstract

**Background:**

The WHO Non-Communicable Diseases Kit (NCDK) was developed to support care for non-communicable diseases (NCDs) in humanitarian settings. Targeting primary healthcare, each kit contains medicines and supplies that are forecasted to meet the needs of 10,000 people for 3 months. This study aimed to evaluate the NCDK deployment process, contents, usage and limitations, and to explore its acceptability and effectiveness among healthcare workers (HCWs) in South Sudan.

**Methods:**

This mixed-method observational study captured data from pre-and-post NCDK deployment. Six data collection tools included: (i) contextual analysis, (ii) semi-structured interviews, in addition to surveys measuring/assessing (iii) healthcare workers’ knowledge about NCDs, and healthcare workers’ perceptions of: (iv) health facility infrastructure, (v) pharmaceutical supply chain, and (vi) NCDK content. The pre- and post-deployment evaluations were conducted in four facilities (October-2019) and three facilities (April-2021), respectively. Descriptive statistics were used for quantitative data and content analysis for open-ended questions. A thematic analysis was applied on interviews findings and further categorized into four predetermined themes.

**Results:**

Compared to baseline, two of the re-assessed facilities had improved service availability for NCDs. Respondents described NCDs as a growing problem that is not addressed at a national level. After deployment, the same struggles were intensified with the COVID-19 pandemic. The delivery process was slow and faced delays associated with several barriers. After deployment, poor communications and the “push system” of inventories were commonly perceived by stakeholders, leading to expiry/disposal of some contents. Despite being out-of-stock at baseline, at least 55% of medicines were found to be unused post-deployment and the knowledge surveys demonstrated a need for improving HCWs knowledge of NCDs.

**Conclusions:**

This assessment further confirmed the NCDK role in maintaining continuity of care on a short-term period. However, its effectiveness was dependent on the health system supply chain in place and the capacity of facilities to manage and treat NCDs. Availability of medicines from alternative sources made some of the NCDK medicines redundant or unnecessary for some health facilities. Several learnings were identified in this assessment, highlighting barriers that contributed to the kit underutilization.

**Supplementary Information:**

The online version contains supplementary material available at 10.1186/s13031-023-00525-w.

## Introduction

The World Health Organization (WHO) has developed standard health kits with essential medicines and medical devices to fulfil various health needs in humanitarian contexts. These kits were created to supply the vulnerable of humanitarian emergencies with dependable and affordable medicines and supplies. Contents of these kits are routinely evaluated and revised by the WHO and partners, responding to changing demands and learning from previous experiences [1]. Unlike the WHO Interagency Emergency Health Kit (IEHK) which provides only few drugs and devices for the management of NCDs, [2] the WHO Non-Communicable Diseases Kit (NCDK) was developed in 2016 to address the growing need of delivering continuity of care in humanitarian settings, as well as a secondary prevention of non-communicable diseases (NCDs) and their associated exacerbations [3].

The Eastern Mediterranean Region (EMR) has a high rate of NCDs and known as a site of numerous major conflicts, hosting many of the world’s refugees. Accordingly, the WHO Regional Office for the Eastern Mediterranean (EMRO) spearheaded the NCDK development. Quantification of the kit content was modelled on the basis of previously collected data, addressing regional prevalence/incidence of major NCDs—namely hypertension and cardiovascular diseases (CVDs), diabetes mellitus (DM), chronic respiratory diseases; asthma/chronic obstructive pulmonary disease (COPD), and selected mental health and neurological conditions [4]. The NCDK is composed of five submodules arranged by NCD type, aligned with the WHO Package of Essential Noncommunicable Disease Interventions (PEN) [5]. The purpose of the kit is to meet a forecasted need of essential medicines and medical devices, at a primary health care (PHC) level, in settings like armed conflicts, epidemics, natural disasters and other emergencies [3]. While the NCDK contains a list of essential medicines for NCDs, its contents may not fully align with the Essential Medicine List of South Sudan, which is specifically tailored to address the diseases that cause the highest mortality and morbidity rates in the country, mainly communicable diseases [6, 7]. Additionally, the NCDK was originally designed to serve populations in the EMR where the prevalence of NCDs is higher [4]. Furthermore, South Sudan and the EMR differ significantly in terms of their healthcare systems, infrastructure, and population needs. The EMR is composed of mostly middle-income countries with established healthcare systems and better infrastructure, while South Sudan is a low-income country that faces significant challenges in providing basic healthcare services due to limited resources and a fragile healthcare system [8, 9]. These differences in healthcare systems, infrastructure, and population needs should be taken into consideration when deploying the NCDK to ensure that the kit items are suitable for the local context. As of today, the NCDK has been deployed in emergency settings outside the WHO EMRO region, including but not limited to countries from Africa Regional Office (AFRO) and South East Asia Regional Office (SEARO). The NCDK is also pre-positioned in the WHO hub in the International Humanitarian City (Dubai, United Arab Emirate) [10]. Several country-level assessments of the NCDK were conducted in collaboration with stakeholders between 2017 and 2021. These assessments were completed in a sample of health facilities in various EMRO countries (Iraq, Syria, Libya, and Yemen) [10]. This assessment aimed to evaluate the NCDK deployment process, contents, usage and limitations outside the EMR, specifically in South Sudan. The assessment also investigated the acceptability and effectiveness of the NCDK among local healthcare workers (HCWs).

## Methods

The assessment used mixed-methods, retrospective and prospective observational approaches to capture data from before-after the NCDK deployment. Data collection tools were piloted and adjusted based on previous learnings, slightly amended between baseline/endline, where relevant [10]. At the outset, a contextual analysis was carried out to summarize background knowledge on the sociopolitical, humanitarian, and health-system considerations that influence the relevance of the kit to the setting.

Qualitative data were obtained through semi-structured interviews targeting key informants from relevant facilities (e.g., Ministry of Health (MoH) and WHO in-country staff). Question guides were tailored to reflect on the assessment timeline nature (baseline/endline) and the scope of NCDK engagement among participants. The interviews included questions to collect feedback about the NCDK ordering and deployment process, NCD management burden, and participants’ recommendations to improve NCD care. The interviews were conducted in person where possible, or remotely via Skype/phone.

A health facility assessment survey was derived from the WHO’s HEARTS Technical Package (a tool that supports ministries of health strengthen CVD management at PHC level) and further aligned with the WHO Package of Essential Noncommunicable Diseases Interventions (WHO-PEN); to collect general information around infrastructure, availability of services, storage capacity, and service delivery [4, 6]. A pharmaceutical supply chain survey was also used to establish background supply chain information and capacity to receive and store the kit content appropriately.

For quantification, an enhanced version of the Dharma tool (a tool developed by WHO EMRO and partners to calculate NCDK remaining stock levels and utilization rates) was used to enable consistency and facilitate comparison of remaining kit items and their utilization rates with other NCDK assessments. At baseline, the general availability status was measured using a 5-point Likert scale—actual quantities were not collected. At endline, specific quantities of the NCDK contents were captured, and the number of occasions where a facility experienced stock-out for seven consecutive days (during the last 90 days).

A short multiple-choice test of up to 20 questions was self-administered to relevant HCWs for 30 minutes (across all cadres/locations) to measure: (A) general NCD knowledge (B) NCDK content knowledge, and (C) perceptions around the NCDK.

Descriptive statistical methods were used for quantitative data, and content analysis was applied to open-ended questions. Qualitative interviews were recorded, transcribed, and then stored in MS Excel. A thematic analysis was then conducted on interviews and quotations. All findings were organized into the following predetermined themes (agreed with WHO/study leads):

- Pre-deployment needs assessment/procurement.
- Logistical capacity, repackaging and delays with kit distribution.
- Kit content analysis, acceptability and relevance to local context.
- Health system and human resources readiness.

Ethical approvals were obtained from the International Rescue Committee (IRC) Institutional Review Board (#00009752) and the local MoH (#MOH/ERB36/2019). An external consultant was hired, trained remotely and mentored by study leads to collect the data. Based on accessibility, the baseline took place in four facilities and in three for endline. The data was collected through physical observations, interviews with relevant staff, and further recorded on paper-based surveys and/or on tablets using the Kobo Toolbox Software, where possible.

## Results

Data was collected using a variety of methods, including contextual analysis (literature review), semi-structured interviews, health facility assessment surveys, pharmaceutical supply surveys, quantification tools, and clinical staff knowledge surveys. Seven key informant interviews were conducted in total, with two at baseline and five at endline. These interviews included two facility managers, a WHO in-country staff member, and representatives from the MoH and the Health Pooled Fund (HPF)—a multi-donor program that collaborates with the government and other partners to enhance the health system and provide essential health services. A total of 40 HCWs (15 at baseline and 25 at endline) responded to the knowledge survey. All facilities (n = 7) were found to be categorized as health clinics or health posts except for two hospitals. Only two facilities were able to complete all assessment tools at baseline and endline. Additional details can be found in Supplementary Material 1. The post-deployment quantification was based on nine months of NCDK utilization. In the following sections, we present our findings using the predetermined themes and the WHO Building Blocks framework where applicable.

### i. Pre-deployment needs assessment and procurement

#### Service delivery

The contextual analysis indicated that NCDs cause high morbidity and mortality in South Sudan, but the health information system (HIS) mainly focuses on infectious diseases, maternal and reproductive health [12]. In 2016, NCDs accounted for 27% of all deaths in South Sudan, with CVDs being the most prominent cause (10%), followed by cancers (7%), chronic respiratory diseases (2%), diabetes (1%), and other NCDs (8%) [12]. A shortage of NCD medications has been observed since 2016–2017, cancer and CVDs may go undiagnosed until recorded as a cause of death [12, 13]. The probability of premature death from NCDs for those aged 30–70 years was 20% in 2020, [14] higher than the Sub-Saharan Africa (SSA) regional average of 13.7% [15]. It is noteworthy that, being a fragile state, the data collection systems in South Sudan are weak and thus the actual figures could be greater in reality. Furthermore, findings from the WHO-led Service Availability and Readiness Assessment (SARA) of healthcare facilities showed significant weaknesses in addressing NCDs, with non-governmental organizations (NGOs) owned facilities having a higher availability of services compared to government owned facilities [16, 17]. The WHO supported the MoH in 2018 in integrating NCD management into PHC through various approaches [18].

At baseline, the interviewees identified NCDs as a growing problem that is not referred to in national policies and strategic documents. An informant stated, *“we do have cases of NCDs but to be very honest with you, it is not a priority, like it is not taken seriously. I mean our biggest issue is usually malaria and maternal health, things like that!”.* The interviews also revealed that conditions such as CVDs, cancers, diabetes, and mental health are of a particular high burden, but donor support focuses mainly on communicable diseases and maternal health. After the NCDK deployment, informants described the same struggles but indicated that they had been exacerbated by the COVID-19 pandemic. Mental health conditions were perceived as increasingly prevalent given the protracted conflict. A national level informant said, *”diabetes and hypertension are well known, but because of the war, everyone here is depressed. Some people grew up seeing horrible acts being committed by soldiers”*. Some informants reported other NCDs (e.g., asthma, diabetes, epilepsy and hypertension) as common clinical problems but to a lesser extent.

### Leadership and governance

South Sudan is the world’s newest nation after splitting from Sudan in 2011 [19]. According to the contextual analysis, public health services are delivered along a four-tier system, ranging between primary and tertiary care levels [20]. The MoH aims to provide policy guidance, leadership, funding, and is responsible for monitoring and evaluation (M&E) activities [20]. At the local level, the country has PHC units to ensure implementation of the Basic Package of Health Services (BPHS) and community participation [20]. Although the other assessment tools did not cover leadership and governance, it was noted that all facilities were heavily reliant on support from NGOs, both at baseline and endline. Additional details can be found in Supplementary Material 1.

### Health workforce

The interviews have shown that some HCWs have left the country due to the negative impact of rainy seasons and floods. This has resulted in a shortage of staff at healthcare facilities. A facility manager said, *“my main focus is more on pushing the government to hire health workers and later start improving the healthcare services”*. The health facility assessment surveys were intended to gather information on the number of HCWs working in the assessed facilities, however, data gaps prevented the collection of this information. Recent statistics demonstrate a severe shortage of trained HCWs categories in South Sudan, with one physician per 65,574 population, and one midwife per 39,088 population [21].

### ii. Logistical capacity, repackaging and delays with kit distribution

The assessment showed that onward deployment of the NCDK after its receipt in Juba was challenging and slow. The kits were initially deployed in Juba, but then had to be moved to other sites, which complicated the process. Key reasons for delaying the kit use included the need for staff training, and also to breakdown communication between various stakeholders. The lack of pre-existing training manuals for the NCDK contents further hindered the speed of its deployment as some HCWs are not familiar with certain kit items. At endline, poor communications around the NCDK orders were reported by most interviewees, including national level informants and facility managers. According to facility staff, this inadequate coordination is a result of the push-based supply chain which is the mainstream of work in the country. Due to lack of M&E systems, poor communications and silo working, stakeholders were not always familiar with what is being delivered to their facilities and sometimes drugs expire and goes for disposal. An interviewee stated *“we are not involved very much in the procurement of supplies, here is the push system. We only receive the drugs and as a result, we have more medicines than we need”*. Notably, some of the medicines in the NCDK were also reported as already or nearly expired on arrival.

### iii. Kit content analysis, acceptability and relevance to local context

#### Service delivery

While the study participants reported that the NCDK was relevant at baseline and appreciated at endline, there were concerns about its long-term sustainability and contribution to strengthening the health system. One informant stated that the MoH estimates that one kit may last longer than projected in the South Sudanese context. The informant said, *“the kit in EMRO region is for 10,000 people for 3 months, whereas in South Sudan, it is estimated that it may provide for 15,000 people over the same period”*. However, this finding must be interpreted in light of the additional data gathered from the other assessment tools, which indicate a lack of training and limited capacity among HCWs, as well as a shortage of clinical guidelines and limited services available for NCDs. Thus, while the NCDK may be viewed as relevant, it must be acknowledged that its effectiveness could be constrained by broader health system challenges. Moreover, cultural factors were expected to act against the kit’s acceptability at baseline interviews, especially for people living with mental health conditions, who are highly stigmatized and often seek traditional healers instead of doctors. Informants also highlighted the issue of HCWs’ perceived scope of practice, as they may request additional pay to treat NCDs. At endline, the NCDK was appreciated, but high-level informants were unsure if they would suggest future orders due to the lower priority given to managing NCDs compared to other diseases. According to stakeholders, facilities should be given the option to adjust the NCDK content according to their local needs. An interviewee said, *“In future, the NCD kit will need to be more context appropriate with specific contents. It may need repackaging for relevant diseases and country guidelines”.* Some of the kit content may be less relevant to the setting and therefore not as effective (such as risperidone, bisoprolol, and hydrochlorothiazide), which are not included in local guidelines and HCWs may not be familiar with it. A facility manager added, *“Type 1 diabetes and insulin are rarely seen and therefore less relevant, but also where it is needed, sufficient capacity may not exist to facilitate its use, as proper cold chain systems are lacking”.*

### Medical products

The post-deployment assessment revealed that facilities were supported by the WHO and other actors who provided donations of NCD medicines in addition to the drugs included in the NCDK, leading to accumulation and ultimately expiration and disposal. Low uptake resulted in a surplus of medications across facilities. At baseline, essential medicines were frequently out-of-stock (OOS) in both facilities, limiting patient care. Although some improvements were observed at endline, stockouts of certain medicines persisted (e.g., amlodipine, isosorbide dinitrate and prednisolone). However, quantification and verification visits indicated that at least 55% of medicines received from the NCDK were still on shelves despite being OOS at the baseline visit. For example, drugs like bisoprolol, hydrochlorothiazide, fluoxetine and beclomethasone were completely untouched as result of the multiple donations, limited community needs and HCWs’ capacity. Additional details can be found in Supplementary Material 2. Informants at the facility level also reported that the medicines received from the NCDK exceeded their needs, which led to the accumulation and expiration of some medicines, particularly fluoxetine, bisoprolol, furosemide, and hypoglycemic agents, due to multiple donations and limited needs. According to one facility manager, “*every other month, we dispose a lot of expired medicine because it is so much more than we need. The MoH has three rooms to keep expired drugs, imagine that!*”, and another facility manager expressed the desire to be able to order the medicines they need from the kit, in the quantities they need, stating, *“If it was possible, I would return some medicines to Juba, e.g., the bisoprolol and furosemide, because we already have them, from UNICEF and IMC (International Medical Corps)”.*

### iv. Health system and human resources readiness

#### Service provision and delivery

Based on the contextual analysis, the population has grown by almost 18% within a decade, reaching 11.2 million at present [22]. The country has a young population with a median age of 19 years, and the largest population group is aged between 15–64 years [23]. The civil war started in December 2013 and the conflict has disproportionately affected women and children [24]. About 50% of health facilities are dilapidated or destroyed, and many lack or have outdated medical and surgical equipment [25]. In addition to displacement, starvation, agricultural crisis, and COVID-19, the conflict has intensified as the pandemic slowed the implementation of peace deals and delivery of aid [26].

Service availability for NCDs was low at the four baseline facilities. When two facilities were re-assessed, their capacity to diagnose (hypertension, diabetes, asthma, COPD and epilepsy), and manage (asthma, COPD, and epilepsy) have improved. However, they were found to have inadequate counseling/education services, a lack of community activities, poor screening/referral practices, according to the facility assessment surveys. A high-level informant stated, *“Right now, some services have stopped completely. Like health education. Nurses are overwhelmed at the outpatient; they cannot do both”.*

### Health workforce

The average overall percentages of the knowledge surveys demonstrated a need for improving HCWs’ capacity. Two prescribers who responded at baseline to NCDK content and clinical questions scored 28% and 27% for both sections, respectively. Non-prescribers (n = 15) had corresponding scores of 24% on NCDK content and 56% on NCD clinical questions. At endline, ten respondents reported prescribing medications to patients with NCDs; they scored 39% on average in the NCDK content, and 68% in the NCD clinical questions. Additional details can be found in Supplementary Material 3. Perceived challenges at baseline included the lack of medicines/supplies and frequent stockouts (most common), HCWs shortages, and low awareness around NCDs among other obstacles. Reported challenges for the HCWs after deployment were mainly related to cultural attitudes and poor awareness among the community, coupled with other health system challenges such as the limited financial and human resources. These findings were further supported by several interviews that revealed similar patterns. A national-level informant noted that “*inadequate staff training on NCDs, particularly at the primary care level, is an issue for effective kit use*”.

### Health information systems

The assessed facilities were found to solely rely on a registry system for record-keeping and store patient files only in paper form. No electronic HIS were found in use, and there were no NCD-specific registers or tracking systems available to enhance the continuity of care. Patient files are consulted only when necessary, and there is no system in place to keep clinical records for each patient to facilitate follow-up. Although stock cards and logbooks for medications and consumables are available, they are not routinely used. No systematic process for needs assessment was observed at both data points (baseline/endline), nor was the existence of a systematic M&E systems in place; a significant concern as it has implications for forecasting pharmaceuticals and health supplies, which are essential for maintaining an effective healthcare system. An informant stated *“NCDs are a growing problem in South Sudan, and not referred to in many documents because country has not had provisions to capture data at NCD level.”*

### Health financing

Although the assessment did not specifically aim to evaluate health financing in the assessed facilities, we collected general data from the literature and the health facility assessment surveys to provide a broader context of the situation. Our findings revealed that the facilities heavily relied on NGOs for support, both at baseline and endline. This was largely due to a lack of sustainable funding sources for medical supplies and staff support, as described by interviewees who reported their complete dependence on donors and humanitarian aid. One interviewee stated, “*we still rely 100% on donors, on humanitarian aid. The country cannot function without donor money*.“ According to the literature, NGOs are responsible for almost 80% of health service delivery in South Sudan [25].

The government spending on health is exceptionally low at just below $27 per capita, which is significantly lower than the SSA regional average of $83.25. Government funding for health is less than 2% of the national budget, and out of pocket spending accounts for around 54% of total health expenditure [27].

## Discussion

The impact of fragile states on NCDs in humanitarian contexts is a crucial consideration. Fragile states are more susceptible to conflict, instability, and economic hardship, which can exacerbate the burden of NCDs [28]. The unique challenges presented by such contexts must be considered to develop effective approaches to addressing NCDs in these settings [29]. Humanitarian emergencies exacerbate factors such as: low prioritization of NCD care, limited implementation of Universal Health Coverage (UHC), inadequate screening and risk assessment practices, the need for national regulations governing NCDs, the role of intersectoral partnerships and collaborations, gaps in public health surveillance, unequal distribution of HCWs, and providers’ limited capacity to address and manage NCDs adequately [30]. In addition to these challenges, the COVID-19 pandemic has had a profound impact on essential health services worldwide [31]. According to WHO, about 90% of countries reported one or more disruptions to essential health services due to the pandemic, including NCD care [32]. As the COVID-19 pandemic has exacerbated the burden of NCDs in fragile states, it is essential to also address its accompanying challenges and implement measures to improve continuity of care and address the needs of people living with NCDs [33].

The findings of this study highlight several key themes related to the challenges of addressing NCDs in the humanitarian context. One important theme is the health component of humanitarian response which has been traditionally centered on the treatment of acute ailments including trauma and infectious diseases [2]. For instance, the IEHK provides a common bundle of pharmaceuticals that includes only a few drugs and devices for the management of NCDs [2]. However, NCDs are extremely common over the world, particularly in low- and middle-income countries (LMICs), and emergencies can raise the likelihood of acute exacerbations and reduce the ability of health systems to respond [2, 34]. The sociopolitical situation, the health systems considerations, the burden of NCDs and the fragile integration of NCD management in PHC all support the relevance of the NCDK in South Sudan. To effectively address the burden of NCDs in such contexts, it is crucial to prioritize the integration of NCD management into PHC. This includes ensuring the availability of essential medicines and supplies at different healthcare system levels, as recommended by the BPHS, which provides a framework for the essential health services that should be provided at these levels, including the medicines that should be available accordingly [35, 36].

Another theme is the importance of continuity of care for NCDs in humanitarian emergencies. A previous NCDK assessment in Yemen and Libya found that the kit has played an important role in maintaining continuity of care for NCDs via providing medicines and supplies for a short-term period, especially when other supply chain solutions were disrupted [10]. However, its effectiveness was dependent on several key components of the health system, including system supply chain, the capacity of facilities and HCWs to manage NCDs, leadership and governance, service delivery, health system financing, medical products and the HIS. Even though similar findings were observed in South Sudan, a number of contextual factors exerted a substantial impact on the utility and efficacy of the NCDK. Notably, the presence of multiple donations of medicines and medical supplies, the relatively lower prioritization of NCDs, the poor M&E systems and the heavy dependence on support from NGOs played pivotal roles in shaping the overall effectiveness and applicability of the NCDK.

A third theme is the need for comprehensive pre-deployment assessments to ensure that the NCDK is relevant and necessary to the local context. Such assessments should include medication and supplies baseline measurement, anticipated patient needs, frequency of future NCDK shipments, supply chain readiness, staff clinical management capacity, coupled with the ability to customize the kit content as per local needs. These factors are essential to ensure the kit is relevant and necessary to the local context. Although assessing the projected need for certain NCDK content is maximally useful if the future NCDK orders can be customizable, such assessment could also support a decision to stop or delay future deployments if the kit’s contents are not fit for purpose in the setting under consideration. Additional rationale for comprehensive pre-deployment needs assessments could include improvements to the effectiveness and efficiency of crisis response, and enhanced trust in the overall humanitarian enterprise [37]. Overall, the challenges faced in managing NCDs in South Sudan are similar to those encountered in other LMICs. These findings underscore the complexity and multifaceted nature of addressing NCDs in humanitarian contexts.

### Study challenges and limitations

Various contextual challenges were encountered during the evaluation process. First, the assessments have been delayed as a result of the lengthy deployment process. It is also expected that supplies from multiple sources could confound the retrospective quantification which was difficult to verify due to poor M&E systems in place. Second, selecting health facilities and conducting data quality assurance was quite challenging due to the complexity of the setting. Because of COVID-19, the planned WHO-led NCD training did not take place, and training of research staff needed to be done remotely, despite technical challenges in the field. It is also plausible that the pandemic might have affected the findings of this study because of the enormous pressure on health systems around the globe, including impacts such as supply chain disruptions, health-seeking behaviors, patient access, and health service utilization [38–40]. Third, the research staff faced additional obstacles at the endline visit as HCWs were on strike over their unpaid incentives, limiting data collection activities due to staff absenteeism. The surveys depended on self-report by respondents, leaving them potentially vulnerable to response and social desirability biases. Nevertheless, it is believed that the information provided through this assessment gave us good information that can be triangulated with findings from similar settings [10].

## Conclusion and recommendations

These findings confirm that the NCDK played an important role in maintaining the continuity of NCD care and providing medicines and supplies for a short-term period [10]. However, its overall impact was dependent on the health system supply chain, the capacity of facilities to manage and treat NCDs, service delivery, health system financing, medical products and the HIS. In some cases, availability of medicines from alternative sources made the medications in the NCDK redundant or unnecessary. Important lessons for the continued use of the kit were recognised despite a number of contextual and methodological constraints of the study (Table 1) [4, 41].

**Table 1:** Recommendation for future kit use

Recommendation	Rational/Description
Establishing a systematic process for pre-deployment needs prior to shipping future kits	To ensure that the kit is relevant and necessary to the local context, gathering information about the emergency situation and working closely with relevant organizations and stakeholders, including the MoH, is essential. This collaborative approach can help to identify the specific needs of the community and support a decision to halt further orders if needs are found to be limited.
Establishing and/or strengthening M&E systems	The provision of M&E tools such as logs and the use of barcode scanners on medicines could support effective utilization of the kit (e.g., pharmacy managers using mobile applications with barcode scanners). However, service implementation may face challenges such as technical issues, lack of training/resources, or resistance to change. Careful assessment and stakeholder collaboration can mitigate these barriers for optimal M&E tool usage.
Work around the push-based supply chain system	Future deployments must take into account that pull-based supply chain systems are generally preferred over push-based systems; multiple donors were found to support facilities with the same medicines. A quicker delivery process is required to ensure efficacy of the kit. It is recommended to incorporate the NCDK deployment system via a common distribution channel (chain). In South Sudan, for instance, it can be done through the Central Medical Store or by utilizing the supply chain of key partners like the Health Pooled Fund (HPF) or United Nations Children’s Fund (UNICEF) for World Bank Project supported sites.
Assigning relevant WHO standard emergency health kits according to local capacity and need	It is suggested to revise the IEHK and reduce its NCD content and aim to utilize it in settings with low NCD burden, low apparent burden and/or places with little capacity to manage NCDs; reserving the NCDK for higher burden areas where the infrastructure and capacity to manage these conditions are better established. However, it is important to acknowledge that not all emergency responses may have access to the NCDK, and in such cases, having adequate NCD medicines in the IEHK can be useful. The IEHK and NCDK should be seen as complementary tools assigned based on local need.
Reviewing the NCDK content	Observations from previous and current assessments of the NCDK suggest a need to review its content. It is recommended to introduce flexibility around the items included in the kit, as well as their quantities and strengths, to align with the BPHS, local essential medicine lists, and tailored to local needs. It would be ideal to organize the NCDK material by level of service delivery, taking into account the capacity and training of HCWs at each level and in accordance with local practices and guidelines.
Reframing the NCDK contents into further sub-categories	It is suggested that the NCDK modules could be reframed by separating out medicines and supplies into further sub-categories by disease (e.g., cardiovascular disease sub-module etc.). Country-specific contexts and needs must be considered to ensure that the sub-categories are recognized and relevant. Considerations for a minimum package of essential mental health services at PHC level should also be made and rolled with comprehensive trainings in the future.
Capacity strengthening of HCWs	The past and present assessments have revealed several gaps in NCD training. As a result, trainings should be implemented before or concurrentlywith the deployment of the NCDK, including periodic refreshers to guarantee HCWs are comfortable and competent in utilizing the kit.
Ensuring the model of NCD service integration is defined prior the deployment of commodities	It is important to note that the approach to NCD service integration may vary depending on the healthcare system and context of each country. Therefore, the model of NCD service integration should be tailored to the specific needs and resources of each country’s health system. For example, setting up a separate NCD clinic is likely to improve the quality of services as health counseling, patient education, and record keeping practices are likely to be improved when provided systematically.
Ensuring quality of service delivery	When deploying the NCDK, it is crucial to ensure availability of guidelines and protocols adopted to the local context accompany the kits at the time of deployment. These resources must be made readily accessible to HCWs to ensure proper utilization of the kits, with systems in place to monitor adherence to them. Furthermore, it is highly recommended to establish systems for capturing health information and patient records before or during the deployment process to ensure that these records are accurately documented and maintained.
Development of an essential medicine list for NCDs at the PHC level	While many essential medicines for NCDs may already be included in the existing national list of medicines, establishing a separate essential medicine list for NCDs at the PHC level can help to ensure that these medicines are readily available in PHC settings and facilitate transition into normal supply chain channels in the future. Moreover, by connecting this list to existing national guidance and training programs, HCWs can be better equipped to properly prescribe and administer these medicines, which can ultimately contribute to better health outcomes for patients with NCDs.

In addition to the short-term benefits of the NCDK, it is important to consider its potential for long-term sustainability and contribution to the strengthening of the national health system. The use of the NCDK has the potential to demonstrate the scale of need for NCD services and can contribute towards the inclusion of required medicines for NCDs in the country’s essential medicines list. It can also lead to the better capture of health information data on NCDs, and thus aid in the strengthening of the national health system for the long-term. Efforts to scale up or replicate NCD programs can further enhance the impact of the NCDK and improve the management of NCDs. Our findings emphasise the critical importance of addressing pre-deployment needs assessments, tailoring kit content to prevalence/disease patterns, developing HCWs’ capacities to manage NCDs, ensuring the availability of adopted guidelines and protocols prior to deployment, donors and actors working in silos, strengthening weak infrastructures, supply chain strategies, and national HIS—all of which have contributed to the underutilization of the NCDK [41, 42].

## Electronic supplementary material

Below is the link to the electronic supplementary material.

Supplementary Material 1

Supplementary Material 2

Supplementary Material 3

## Data Availability

Additional data and materials used and/or analyzed during the current study are available from the corresponding author (LK) on reasonable request.

## Abbreviations

ACEIs: Angiotensin-converting enzyme inhibitors
AFRO: WHO Africa Regional Office
BPHS: Basic Package of Health Services
CCBs: Calcium Channel Blockers
COPD: Chronic Obstructive Pulmonary Disease
CVDs: Cardiovascular Diseases
DM: Diabetes Mellitus
EMR: Eastern Mediterranean Region
EMRO: WHO Regional Office for the Eastern Mediterranean
HCWs: Healthcare Workers
HIS: Heath Information System
HPF: Health Pooled Fund
IEHK: Interagency Emergency Health Kit
LMICs: Low- and Middle-Income Countries
M&E: Monitoring and Evaluation
MoH: Ministry of Health
NCDK: WHO Non-Communicable Diseases Kit
NCDs: Non-Communicable Diseases
NGOs: Non-Governmental Organizations
OOS: Out-of-Stock
PHC: Primary Health Care
SARA: Service Availability and Readiness Assessment
SEARO: South East Asia Regional Office
SSA: Sub-Saharan Africa
WHO: World Health Organization
WHO PEN: WHO Package of Essential Noncommunicable Disease Interventions
