# Supplementary material for: Assessment of the WHO non-communicable diseases kit for humanitarian emergencies in South Sudan: a retrospective, prospective, observational study
Source: Confl Health. 2023 Jun 5;17:27. doi: 10.1186/s13031-023-00525-w (PMC10241119; doi:10.1186/s13031-023-00525-w)
Supplement: Supplementary file 1 — Supplementary Material 1 [file 13031_2023_525_MOESM1_ESM.docx]

**Supplementary Material 1: Health Facility Assessment Tables**

### Table 1. Baseline Infrastructural Capacity

| **Health Facility**  **(HF)** | **Baseline** | | | | | | | | | | |
| --- | --- | --- | --- | --- | --- | --- | --- | --- | --- | --- | --- |
|  | **Type/level of health facility** | | **Facility catchment population (reported by HF)** | **supported by other organizations** | **Electricity** | **Storage space capacity** | **Temperature in storage space** | **Control of temperature in storage space** | **Sufficient capacity for cold chain** | |  |
| Bentiu Hospital | Hospital | | 50,000 | Yes | Yes | Very sufficient | (26°C - 29°C) | Unknown | Somewhat sufficient | |  |
| Malakal PHCC^a^ | Health Clinic/Post | | Unknown | Yes | Yes | Sufficient | Unknown (no thermometer) | Unknown | Somewhat sufficient | |  |
| Kotor PHCC | Health Clinic/Post | | 30,000 | Yes | Yes | Very sufficient | (26°C - 29°C) | No | Somewhat sufficient | |  |
| Munuki PHCC | Health Clinic/Post | | 62,000 | Yes | Yes | Somewhat sufficient | Unknown (no thermometer) | Unknown | Somewhat sufficient | |  |
| **Health Facility** | **Diagnostic tests for patients without symptoms for Diabetes** | **Diagnostic tests for patients without symptoms for Hypertension** | **Guidelines for the management of Diabetes** | **Guidelines for the management of Hypertension** | **Guidelines for the management of Asthma & COPD^b^** | **Patients diagnosed with diabetes able to continue follow-up/treatment** | **Patients diagnosed with hypertension able to continue follow-up/treatment** | **Specific NCD^c^ registers for consultations** | **Appointment system for NCD patients** | **Referral criteria** |  |
| Bentiu Hospital | Unknown | Unknown | Unknown | Unknown | Unknown | Yes | Yes | No | No | Yes |  |
| Malakal PHCC | Unknown | No | Unknown | Yes (South Sudan Ministry of Health) | Yes (South Sudan Ministry of Health) | Unknown | Unknown | Yes | Yes | No |  |
| Kotor PHCC | Unknown | Unknown | Unknown | Unknown | Unknown | No | No | Unknown | Unknown | No |  |
| Munuki PHCC | No | No | No | No | Unknown | No | No | No | No | No |  |

^a^ PHCC, Primary healthcare center

^b^ COPD, Chronic obstructive pulmonary disease

^c^ NCD, Noncommunicable disease

### Table 2. Endline Infrastructural Capacity

| **Health Facility** | **Endline** | | | | | | | |
| --- | --- | --- | --- | --- | --- | --- | --- | --- |
|  | **Electricity** | **Water supply** | **Supported by other organizations** | **Access to computer** | **Cellular network to support internet access** | **Laboratory services available** | **Time to receive basic blood tests results** |  |
| Bentiu Hospital | Yes | Yes | Yes | Yes | Always | Yes | Same day |  |
| Malakal PHCC^a^ | Yes | Yes | Yes | No | Always | Yes | Same day |  |

^a^ PHCC, Primary healthcare center

### Table 3. Baseline and endline Priority NCD services offered

| **Health Facility** | **Baseline** | | | | | | | | | |
| --- | --- | --- | --- | --- | --- | --- | --- | --- | --- | --- |
|  | **Hypertension** | | **CVDs^b^** | | **Diabetes** | | **Asthma/COPD^c^** | | **Epilepsy** | |
|  | **Diagnosis** | **Treatment /follow-up** | **Diagnosis** | **Treatment /follow-up** | **Diagnosis** | **Treatment /follow-up** | **Diagnosis** | **Treatment /follow-up** | **Diagnosis** | **Treatment /follow-up** |
| Bentiu Hospital | No | Yes | No | No | No | Yes | No | No | No | No |
| Malakal PHCC^a^ | Yes | Limited | No | No | Limited | Limited | Yes | Yes | Yes | Yes |
| Kotor PHCC | No | Yes | No | No | Limited | No | No | No | No | No |
| Munuki PHCC | Yes | Limited | No | No | Yes | No | No | No | No | No |
|  | **Endline** | | | | | | | | | |
| Bentiu Hospital | Yes | Yes | No | No | Yes | Yes | Yes | Yes | Yes | Yes |
| Malakal PHCC | Yes | Yes | No | No | Yes | Yes | No | No | No | Yes |

^a^ PHCC, Primary healthcare center

^b^ CVDs, Cardiovascular diseases

^c^ COPD, Chronic obstructive pulmonary disease

### Table 4. Patient Counselling & Education - Endline

| **Health Facility** | **Patient Counselling & Education** | | | | | | | |
| --- | --- | --- | --- | --- | --- | --- | --- | --- |
|  | **Smoking** | **Diet** | **Alcohol consumption** | **Physical activity** | **Family members (on smoking, diet, alcohol consumption, physical activity)** | **Cardiovascular risk assessment** | **Self-management of diabetes** | **Self-administration of insulin** |
| Malakal PHCC^a^ | Yes | Yes | Yes | Yes | No | No | No | No |
| Bentiu State Hospital | No | Yes | Yes | No | No | No | Yes | Yes |

^a^ PHCC, Primary healthcare center

### Table 5. Health Education Materials - Endline

| **Health Facility** | **Health Education Materials** | | | | | |
| --- | --- | --- | --- | --- | --- | --- |
|  | **Smoking** | **Diet** | **Alcohol consumption** | **Physical activity** | **Hypertension** | **Diabetes** |
| Malakal PHCC^a^ | No | No | No | No | No | No |
| Bentiu State Hospital | No | No | No | No | No | No |

^a^ PHCC, Primary healthcare center

### Table 6. Other services offered - Endline

| **Health Facility** | **Community activities to support NCD^b^ services** | **Testing patients without symptoms for diabetes** | **Testing patients without symptoms for hypertension** | **Patients pay for consultations** | **Payment for diagnostic tests** | **Amount of consultations for patients changed since the reception of the NCDK^c^** |  |
| --- | --- | --- | --- | --- | --- | --- | --- |
|  |  |  |  |  |  |  |  |
| Malakal PHCC^a^ | No | No | No | No, consultations are provided free of charge | No, diagnostic tests are provided free of charge | No change |  |
| Bentiu State Hospital | No | Unknown | Unknown | No, consultations are provided free of charge | No, diagnostic tests are provided free of charge | No change |  |
| **Health Facility** | **Ambulance** | **If no, can a patient transfer by ambulance be arranged?** | **Patients’ referral** | **Appointment system for NCD patients** | **Record of patient visits** | |  |
| Malakal PHCC | No | Never | Unknown | No | Yes, records kept for certain types of visits | |  |
| Bentiu State Hospital | Yes, functional | | Yes | No | Yes, records kept for all visits | |  |

^a^ PHCC, Primary healthcare center

^b^ NCD, Non-communicable disease

^c^ NCDK, Non-communicable disease kit

### Table 7. Availability of Guidelines - Endline

| **Health Facility** | **Clinical guidelines** | **NCD**^b^ **risk factors guidelines** | **Familiarity with WHO PEN**^c^ **Guidelines** | **Familiarity with (mhGAP)**^d^ **Guidelines?** |
| --- | --- | --- | --- | --- |
|  |  |  |  |  |
| Malakal PHCC^a^ | No guidelines available | No guidelines available | Yes | No |
| Bentiu State Hospital | Hypertension Diabetes Asthma & COPD Stroke Epilepsy Depression & Anxiety Psychosis  (From MoH) | No guidelines available | Yes | Yes |

^a^ PHCC, Primary healthcare center

^b^ NCD, Non-communicable disease

^c^ WHO-PEN, WHO Package of Essential Noncommunicable Disease Services

^c^ mhGAP, Mental Health Gap Action

### Table 8. Record Systems - Endline

| **Health Facility** | **Records Keeping** | **Records kept electronically or on paper** | **Patient files retrieval** | **Registers specifically for NCD^b^ consultations** | **Clinical records kept for each individual patient to facilitate follow-‘up** | **Call and recall system (tracing of defaulters) of patients with NCD to improve continuity of care** | **Facility has a stock card or logbooks for medications** | **Facility has a stock card or logbooks for consumables** |
| --- | --- | --- | --- | --- | --- | --- | --- | --- |
|  |  |  |  |  |  |  |  |  |
| Malakal PHCC^a^ | Registry system only | Paper only | Yes, patient files consulted, but only when necessary | No | No | No | Yes, but not used routinely | Yes, but not used routinely |
| Bentiu State Hospital | Registry system only | Paper only | Yes, patient files consulted, but only when necessary | No | No | No | Yes, but not used routinely | Yes, but not used routinely |

^a^ PHCC, Primary healthcare center

^b^ NCD, Non-communicable disease

### Table 9. Nongovernmental organizations Supporting Facilities

| **Health Facility** | **Baseline** |
| --- | --- |
| Malakal PHCC^a^ | International Medical Corps (IMC) |
| Munuki PHCC | Health link South Sudan, World Vision, Johns Hopkins Program for International Education in Gynecology and Obstetrics (JHPIEGO), United Nations Development Programme (UNDP), Catholic Organisation for Relief and Development Aid (Cordaid), World Health Organization (WHO) |
| Kotor PHCC | Health Link South Sudan, World Vision, JHPIEGO, Intrahealth International, UNDP, Catholic Relief Services (CRS), United Nations Population Fund (UNFPA), International Committee of the Red Cross (ICRC) |
| Bentiu Hospital | Cordaid |
| **Health Facility** | **Endline** |
| Malakal PHCC | Médecins Sans Frontières (MSF/Doctors Without Borders), United Nations Children’s Fund (UNICEF), IMC, WHO |
| Bentiu State Hospital | Health Pooled Fund (HPF), Cooperative for Assistance and Relief Everywhere (CARE), Cordaid, and private donors |

^a^ PHCC, Primary healthcare center
