# Supplementary material for: Assessment of the WHO non-communicable diseases kit for humanitarian emergencies in South Sudan: a retrospective, prospective, observational study
Source: Confl Health. 2023 Jun 5;17:27. doi: 10.1186/s13031-023-00525-w (PMC10241119; doi:10.1186/s13031-023-00525-w)
Supplement: Supplementary file 2 — Supplementary Material 2 [file 13031_2023_525_MOESM2_ESM.docx]

**Supplementary Material 2: Quantification of Kit Content Utilization According to Therapeutic Areas/Diseases**

At baseline, the general availability status was collected using the following likeart scale [always available (no stock outs), mostly available [(stock outs occur, but rarely), sometimes available (stock outs occur every 1-2 months), rarely available (stock outs occur regularly), never (has not been available at all for >3 months)]. At endline, data were captured around specific quantities of the NCDK contents and the number of occasions where a facility experienced stock-out for seven consecutive days (during the last 90 days). The following sections/tables details the quantification data as per their NCD respective area.

#### Asthma/Chronic obstructive pulmonary disease

At baseline, Steroidal and Salbutamol inhalers, as well as Systematic Steroids were all found out of stock (OOS) in both facilities. The general status of their availability in Bentiu Hospital was extremely poor as they did not have access to such medicines for more than three months. The situation was similar in Malakal PHCC as the access was reported to be extremely limited. Hence, omitting the ability of both facilities to treat asthma and chronic obstructive pulmonary disease (COPD). Only Bentiu Hospital had access to peak flow meters. At endline, the Prednisolone received from kits was found OOS on the assessment day in both facilities. Of the 1,500 Salbutamol inhalers received in each facility, it was found to be OOS in Malakal and only 46 inhalers were found in Bentiu. Beclomethasone inhalers available on the assessment day in Malakal and Bentiu were 300 and 116 inhalers, respectively. 40% and 77% of the received Beclomethasone in Malakal and Bentiu was utilized. (For more information, please see Tables 1&2)

##### Table 1. Baseline Quantification of Pharmaceuticals to treat Asthma/COPD

| **Asthma / COPD** | | **Baseline (Oct 2019)** | |
| --- | --- | --- | --- |
| **Health facility** | **Pharmaceutical class /specific drug name where relevant** | **Availability on assessment day** | **General availability** |
| Bentiu Hospital | Steroid inhaler | OOS* | Never (has not been available at all for >3 months) |
|  | Systemic steroids | OOS | Never (has not been available at all for >3 months) |
|  | Salbutamol inhaler | OOS | Never (has not been available at all for >3 months) |
| Malakal PHCC | Steroid inhaler | Available today | Rarely available (stockouts occur regularly) |
|  | Systemic steroids | Available today | Rarely available (stockouts occur regularly) |
|  | Salbutamol inhaler | OOS | Sometimes available (stockouts occur every 1-2 months) |

*Out of stock (OOS)

##### Table 2. Endline Quantification of Pharmaceuticals to treat Asthma/COPD

| **Asthma / COPD** | | **Kit Deployment (July 2020)** | | | | **Endline (April 2021)** | | | |
| --- | --- | --- | --- | --- | --- | --- | --- | --- | --- |
| **Health Facility (HF)** | **Pharmaceutical class /specific drug name** | **Kit item** | **Strength** | **Unit** | **Quantity received from kits** | **Availability on assessment day** | **Actual AMC*** | **HF reported AMC** | **Number of stock-outs occasions** |
| Bentiu Hospital | Steroid inhaler | Beclomethasone | 250 mcg inhaler/dose | inhaler/dose, 200 doses | 500 | 116 | 43 | Unknown | 0 |
|  | Systemic steroids | Prednisolone | 5mg | tabs | 7,700 | OOS** | N/A*** |  | Unknown |
|  | Salbutamol inhaler | Salbutamol metered dose inhaler (aerosol) | 100 mcg (as sulfate)/200 doses | oral inh. | 1,500 | 46 | 162 |  | 0 |
| Malakal PHCC | Steroid inhaler | Beclomethasone | 250 mcg inhaler/dose, 200 doses | inhaler/dose, 200 doses | 500 | 300 | 22 |  | 0 |
|  | Systemic steroids | Prednisolone | 5mg | tabs | 7,700 | OOS | N/A |  | Unknown |
|  | Salbutamol inhaler | Salbutamol metered dose inhaler (aerosol) | 100 mcg (as sulfate)/200 doses | oral inh. | 1,500 | OOS | N/A |  |  |

*Average Monthly Consumption

**Out of stock (OOS)

*** Not applicable (N/A)

#### Cardiovascular diseases

In Bentiu Hospital, all essential pharmaceutical classes to treat CVDs were OOS on the baseline assessment day except for Calcium channel blockers (CCBs). The general status of access to these drugs was extremely poor as they were not in stock for more than three months. In Malakal PHCC, ACE inhibitors (ACEIs) and Nitrates were found OOS on the assessment day, with ACEIs being rarely available and Nitrates being mostly available. However, stockouts seems to happen regularly. During the endline consultant visit, both facilities were OOS for Amlodipine and Isosorbide dinitrates, and both facilities showed scarcity in Glyceryl trinitrate supplies (less than 50 units were found in both settings). Acetylsalicylic acid was consumed by 75% in Malakal yet found OOS in Bentiu. Furosemide stocks dropped by 80% in Bentiu and by 28% in Malakal. However, Bisoprolol was left untouched in Malakal and only 33% of the received stocks were used in Bentiu. The same was also seen with Enalapril since both facilities have only used 10% to 20% of the supply received. Hydrochlorothiazide was untouched in Malakal and consumed by 28% in Bentiu. (For more information, please see Tables 3&4)

##### Table 3. Baseline Quantification of Pharmaceuticals to treat CVDs

| **CVDs** | | **Baseline (Oct 2019)** | |
| --- | --- | --- | --- |
| **Health Facility** | **Pharmaceutical class /specific drug name where relevant** | **Availability on assessment day** | **General availability** |
| Bentiu Hospital | Acetylsalicylic acid tablets | OOS* | Never (has not been available at all for >3 months) |
|  | Calcium channel blockers | Available | Rarely available (stockouts occur regularly) |
|  | Beta-blocker | OOS | Never (has not been available at all for >3 months) |
|  | ACE inhibitor | OOS | Never (has not been available at all for >3 months) |
|  | Loop diuretics | OOS | Rarely available (stockouts occur regularly) |
|  | Nitrates | OOS | Never (has not been available at all for >3 months) |
|  | Thiazide diuretic | OOS | Never (has not been available at all for >3 months) |
| Malakal PHCC | Acetylsalicylic acid tablets | Available | Mostly available (stockouts occur, but rarely) |
|  | Calcium channel blockers | Available | Mostly available (stockouts occur, but rarely) |
|  | Beta-blocker | Available | Sometimes available (stockouts occur every 1-2 months) |
|  | ACE inhibitor | OOS | Rarely available (stockouts occur regularly) |
|  | Loop diuretics | Available | Sometimes available (stockouts occur every 1-2 months) |
|  | Nitrates | OOS | Mostly available (stockouts occur, but rarely) |
|  | Thiazide diuretic | Available | Sometimes available (stockouts occur every 1-2 months) |

*Out of stock (OOS)

##### Table 4. Endline Quantification of Pharmaceuticals to treat CVDs

| **Asthma / COPD** | | **Kit Deployment (July 2020)** | | | | **Endline (April 2021)** | | | |
| --- | --- | --- | --- | --- | --- | --- | --- | --- | --- |
| **Health Facility (HF)** | **Pharmaceutical class /specific drug name** | **Kit item** | **Strength** | **Unit** | **Quantity received from kits** | **Availability on assessment day** | **Actual AMC*** | **HF reported AMC** | **Number of stock-outs occasions** |
| Bentiu Hospital | Acetylsalicylic acid tablets | Acetylsalicylic acid | 100mg | tabs | 22,000 | OOS** | N/A*** | Unknown | Unknown |
|  | Calcium channel antagonist | Amlodipine | 5 mg (as maleate, mesylate or besylate) | tabs | 15,000 |  | N/A |  |  |
|  | Beta-blocker | Bisoprolol | 5mg | tabs | 15,000 | 10,080 | 547 | - | - |
|  | ACE inhibitor | Enalapril | 5 mg (as hydrogen maleate) | tabs | 15,000 | 12,000 | 333 | Unknown | 0 |
|  | Loop diuretics | Furosemide | 40mg | tabs | 4,000 | 800 | 356 |  | 0 |
|  | Nitrates | Glyceryl trinitrate | 0.5 mg | (sublingual) tab | 2,900 | 21 | 320 |  | 0 |
|  | Nitrates | Isosorbide Dinitrate | 5 mg | (sublingual) tab | 11,800 | OOS | N/A |  | Unknown |
|  | Thiazide diuretic | Hydrochlorothiazide | 25mg | tabs | 12,000 | 8,700 | 367 |  | 0 |
| Malakal PHCC | Acetylsalicylic acid tablets | Acetylsalicylic acid | 100mg | tabs | 22,000 | 5,460 | 1838 | 10,000 | 0 |
|  | Calcium channel antagonist | Amlodipine | 5 mg (as maleate, mesylate or besylate) | tabs | 15,000 | OOS | N/A | Unknown | Unknown |
|  | Beta-blocker | Bisoprolol | 5mg | tabs | 15,000 | 15,000 | 0 |  | 0 |
|  | ACE inhibitor | Enalapril | 5 mg (as hydrogen maleate) | tabs | 15,000 | 13,500 | 167 |  | 0 |
|  | Loop diuretics | Furosemide | 40mg | tabs | 4,000 | 2,900 | 122 |  | 0 |
|  | Nitrates | Glyceryl trinitrate | 0.5 mg | (sublingual) tab | 2,900 | 28 | 319 |  | 0 |
|  | Nitrates | Isosorbide Dinitrate | 5 mg | (sublingual) tab | 11,800 | OOS | N/A |  | Unknown |
|  | Thiazide diuretic | Hydrochlorothiazide | 25mg | tabs | 12,000 | 12,000 | 0 |  | 0 |

*Average Monthly Consumption

**Out of stock (OOS)

*** Not applicable (N/A)

#### Diabetes and Hypothyroidism

For hypothyroidism treatment, Levothyroxine was found to be OOS in both facilities at baseline. For diabetes, all listed classes were also found OOS except for Metformin. The general status of reported availability in Bentiu was extremely poor and slightly better in Malakal as these drugs are sometimes made available through donors. However, regular stockouts occur every one to two months in Malakal. At endline, the quantity of Metformin received from kits were used by 47% and 60% in Bentiu and Malakal, respectively. Glibenclamide stock was also consumed in a similar manner by 47% and 38% in the respective facilitates. Though no Insulins were ordered from the kit, all surveyed insulins were found OOS in both facilitates. Metformin and Glibenclamide were reported to be accessible in both facilities with no events of OOS reports over the last 90 days (before the visit). Levothyroxine was found OOS in both facilities at endline. Due to poor documentation, the number of reported OOS occasions for the latter medicine was unknown for both settings. (For more information, please see Tables 5&6)

##### Table 5. Baseline Quantification of Pharmaceuticals to treat Diabetes and Hypothyroidism

| **Diabetes and Hypothyroidism** | | **Baseline (Oct 2019)** | |
| --- | --- | --- | --- |
| **Health Facility** | **Pharmaceutical class /specific drug name where relevant** | **Availability on assessment day** | **General availability** |
| Bentiu Hospital | Sulfonylurea | OOS | Never (has not been available at all for >3 months) |
|  | Levothyroxine sodium | OOS | Never (has not been available at all for >3 months) |
|  | Metformin | Available | Rarely available (stockouts occur regularly) |
|  | Intermediate or long-acting insulin | OOS | Never (has not been available at all for >3 months) |
|  | Intermediate or long-acting insulin | OOS | Never (has not been available at all for >3 months) |
|  | Short or rapid-acting insulin | OOS | Never (has not been available at all for >3 months) |
| Malakal PHCC | Sulfonylurea | OOS | Never (has not been available at all for >3 months) |
|  | Levothyroxine sodium | OOS | Sometimes available (stockouts occur every 1-2 months) |
|  | Metformin | Available | Sometimes available (stockouts occur every 1-2 months) |
|  | Intermediate or long-acting insulin | OOS | Sometimes available (stockouts occur every 1-2 months) |
|  | Intermediate or long-acting insulin | OOS | Sometimes available (stockouts occur every 1-2 months) |
|  | Short or rapid-acting insulin | OOS | Sometimes available (stockouts occur every 1-2 months) |

*Out of stock (OOS)

##### Table 6. Endline Quantification of Pharmaceuticals to treat Diabetes and Hypothyroidism

| **Diabetes and Hypothyroidism** | | **Kit Deployment (July 2020)** | | | | **Endline (April 2021)** | | | |
| --- | --- | --- | --- | --- | --- | --- | --- | --- | --- |
| **Health Facility (HF)** | **Pharmaceutical class /specific drug name** | **Kit item** | **Strength** | **Unit** | **Quantity received from kits** | **Availability on assessment day** | **Actual AMC*** | **HF reported AMC** | **Number of stock-outs occasions** |
| Bentiu Hospital | Sulfonylurea | Glibenclamide | 5 mg | tabs | 26,000 | 13,800 | 1,356 | Unknown | 0 |
|  | Levothyroxine sodium | Levothyroxine sodium | 100 mcg (sodium salt) | tabs | 4,100 | OOS** | N/A*** |  | Unknown |
|  | Metformin | Metformin | 500mg | tabs | 60,000 | 31,900 | 3,122 |  | 0 |
|  | Intermediate or long-acting insulin | Human Insulin NPH | 100 IU/ml, 10 ml | vial | Not ordered | OOS | N/A |  | Unknown |
|  | Intermediate or long-acting insulin | Human Insulin Mix 70/30 | 100IU/ml, 10 ml | vial |  |  | N/A | - |  |
|  | Short or rapid-acting insulin | Human Insulin R | 100 IU/ml, 10 ml | vial |  |  | N/A | Unknown |  |
|  | Glycogenolytic agents | Glucagon | 1 mg/ mL. | amp |  |  | N/A |  |  |
| Malakal PHCC | Sulfonylurea | Glibenclamide | 5 mg | tabs | 26,000 | 16,000 | 1,111 |  | 0 |
|  | Levothyroxine sodium | Levothyroxine sodium | 100 mcg (sodium salt) | tabs | 4,100 | OOS | N/A |  | Unknown |
|  | Metformin | Metformin | 500mg | tabs | 60,000 | 23,900 | 4,011 |  | 0 |
|  | Intermediate or long-acting insulin | Human Insulin NPH | 100 IU/ml, 10 ml | vial | Not ordered | OOS | N/A |  | Unknown |
|  | Intermediate or long-acting insulin | Human Insulin Mix 70/30 | 100IU/ml, 10 ml | vial |  |  | N/A |  |  |
|  | Short or rapid-acting insulin | Human Insulin R | 100 IU/ml, 10 ml | vial |  |  | N/A |  |  |
|  | Glycogenolytic agents | Glucagon | 1 mg/ mL. | amp |  | - | N/A | - | - |

*Average Monthly Consumption

**Out of stock (OOS)

*** Not applicable (N/A)

#### Mental health conditions

No data regarding the availability of anti-psychotics, carbamazepine or other general anti-epileptic medications was reported at baseline. However, antidepressants were reported to be OOS in Bentiu on the assessment day yet available in Malakal. The latter facility reported that anti-depressants are mostly accessible in general but stockouts occur rarely. The overall general availability of the other drugs/classes were limited due to poor documentation mechanisms. At endline, carbamazepine, risperidone, and sodium valproate 500mg in Bentiu Hospital were found to be all OOS. On the contrary, only risperidone and sodium valproate 500 mg were found OOS in Malakal. The latter facility did not use the received carbamazepine since its stock dropped by only three percent during the last nine months. For fluoxetine, while the received stock was left untouched in Malakal PHCC, excess amounts were found in Bentiu indicating accumulation of the medicine. For the received sodium valproate 200mg, the stock was used by a little bit more than 40% in both facilities. Stockouts of fluoxetine and sodium valproate 200mg were not experienced in both facilities, and was unknown for risperidone in Malakal, carbamazepine and sodium valproate 500mg in Bentiu. (for more information, please see Tables 7&8)

##### Table 7. Baseline Quantification of Pharmaceuticals to treat Mental Health conditions

| **Mental Health** | | **Baseline (Oct 2019)** | |
| --- | --- | --- | --- |
| **Health Facility** | **Pharmaceutical class /specific drug name where relevant** | **Availability on assessment day** | **General availability** |
| Bentiu Hospital | Carbamazepine/General anti-epileptic medication | N/A* | N/A |
|  | General antidepressant medications | OOS** | Never (has not been available at all for >3 months) |
|  | General anti-psychotic medication/Risperidone | N/A | N/A |
|  | General anti-epileptic medication | OOS | Never (has not been available at all for >3 months) |
| Malakal PHCC | Carbamazepine/General anti-epileptic medication | N/A | N/A |
|  | General antidepressant medications | Available | Mostly available (stockouts occur, but rarely) |
|  | General anti-psychotic medication/Risperidone | N/A | N/A |
|  | General anti-epileptic medication | OOS | Rarely available (stockouts occur regularly) |
|  | General anti-epileptic medication | OOS | Rarely available (stockouts occur regularly) |

*Data not available

**Out of stock (OOS)

##### Table 8. Endline Quantification of Pharmaceuticals to treat Mental Health

| **Mental Health** | | **Kit Deployment (July 2020)** | | | | **Endline (April 2021)** | | | |
| --- | --- | --- | --- | --- | --- | --- | --- | --- | --- |
| **Health Facility (HF)** | **Pharmaceutical class /specific drug name** | **Kit item** | **Strength** | **Unit** | **Quantity received from kits** | **Availability on assessment day** | **Actual AMC*** | **HF reported AMC** | **Number of stock-outs occasions** |
| Bentiu Hospital | Carbamazepine/General anti-epileptic medication | Carbamazepine | 100mg | tabs | 3,200 | OOS** | N/A*** | Unknown | Unknown |
|  | General antidepressant medications | Fluoxetine | 20mg | tabs | 5,000 | 9,000 | N/A |  | 0 |
|  | General anti-psychotic medication/Risperidone | Risperidone | 2mg | tabs | 400 | OOS | N/A | - | - |
|  | General anti-epileptic medication | Sodium Valproate | 500mg | tab. (enteric coated) | 1,900 |  | N/A | Unknown | Unknown |
|  | General anti-epileptic medication | Sodium Valproate | 200mg | scored tabs | 3,200 | 1,700 | 167 |  | 0 |
| Malakal PHCC | Carbamazepine/General anti-epileptic medication | Carbamazepine | 100mg | tabs | 3,200 | 3,100 | 11 |  | 0 |
|  | General antidepressant medications | Fluoxetine | 20mg | tabs | 5,000 | 5,000 | 0 |  | 0 |
|  | General anti-psychotic medication/Risperidone | Risperidone | 2mg | tabs | 400 | OOS | N/A | - | Unknown |
|  | General anti-epileptic medication | Sodium Valproate | 500mg | tab. (enteric coated) | 1,900 | OOS | N/A | Unknown | 0 |
|  | General anti-epileptic medication | Sodium Valproate | 200mg | scored tabs | 3,200 | 1,800 | N/A |  | 0 |

*Average Monthly Consumption

**Out of stock (OOS)

*** Not applicable (N/A)

#### General equipment/supplies for NCDs

A good level of access was observed in both facilities at baseline for general equipment and supplies. The general status of surveyed items’ availability was also reported to be acceptable, at least for the past three months prior the assessment day. Only peak flow meters were found OOS in Malakal, as these have not been available/functional for more than three months prior to baseline visit. Since the deployed number of sub-modules 1c (renewable) and 1d (equipment supplies) were unknown for both facilities, the exact quantities received from the Kit were limited. Although no orders for the cold chain sub-module were made, insulin syringes were found OOS in Bentiu, and a total of 3,200 syringes were found in Malakal on the endline assessment day. The latter facility reported no occasions of insulin syringes stockouts, but it was unknown in Bentiu. Limited access to urine test strips was observed in both facilities (200 versus 500 units in Malakal). For Blood Glucose strips, a total of 700 and 500 strips were found in Bentiu and Malakal, respectively. Blood lancets were accessible in higher amounts for both facilities with 2,000 lancets in Bentiu and 3,000 for Malakal. Both settings had access to two peak flow meters which were received from the kit and was found functional/available on the assessment day. similar observation was seen in each facility for the peak flow meter disposable mouth pieces (five pieces in each setting). Eleven glucometers were also found in each facility despite the original kit containing only three per consignment, which indicated other supplies were coming from different donors. Similarly, extra number of sphygmomanometers were also seen in both facilities. The remaining supplies were almost found in its originally received quantities and all equipment were functional in both settings. Stockouts of these items were never reported in the most recent 90 days before the visit for both facilitates. (For more information, please see Tables 9&10).

##### Table 9. Baseline Quantification of general equipment/supplies available for NCDs on the assessment day

| **General Equipment/Supplies** | | **Baseline (Oct 2019)** | |
| --- | --- | --- | --- |
| **Health Facility** | **Equipment/supply** | **Availability on assessment day** | **General availability** |
| Bentiu Hospital | Blood test strips | Available | Available/functional only for the past 1-2 months |
|  | Blood lancet | Available | Always available/functional during past 3 months or longer |
|  | Glucometer | Available | Available/functional only for the past 1-2 months |
|  | Peak flow meters | Available | Available/functional only for the past 1-2 months |
|  | Stethoscope | Available | Always available/functional during past 3 months or longer |
|  | Sphygmomanometer | Available | Available/functional only for the past 1-2 months |
|  | Measuring tape | Available | Always available/functional during past 3 months or longer |
|  | Thermometer | Available | Always available/functional during past 3 months or longer |
| Malakal PHCC | Blood test strips | Available | Always available/functional during past 3 months or longer |
|  | Blood lancet | Available | Always available/functional during past 3 months or longer |
|  | Glucometer | Available | Always available/functional during past 3 months or longer |
|  | Peak flow meters | OOS* | Has NOT been available/functional for more than 3 months |
|  | Stethoscope | Available | Always available/functional during past 3 months or longer |
|  | Sphygmomanometer | Available | Always available/functional during past 3 months or longer |
|  | Measuring tape | Available | Always available/functional during past 3 months or longer |
|  | Thermometer | Available | Always available/functional during past 3 months or longer |

*Out of stock (OOS)

##### Table 10. Endline quantification of general equipment/supplies available for NCDs

|  | **Kit Deployment (July 2020)** | | | **Endline (April 2021)** | | | |
| --- | --- | --- | --- | --- | --- | --- | --- |
| **Health Facility (HF)** | **Kit item** | **Sub-module** | **Quantity received from Kits** | **Quantity available (assessment day)** | **Status** | **HF reported AMC*** | **Number of stock-outs occasions** |
| Bentiu Hospital | Insulin syringes, 3 pieces, IU-100 | 1b | Not ordered | OOS** | - | Unknown | Unknown |
|  | Urine test strips for ketones, glucose and protein | 1c | Unknown | 200 | Available AND Functional |  | 0 |
|  | Blood Glucose strips | 1d | Unknown | 700 |  |  | 0 |
|  | Lancet, blood, ster, disp, | 1d | Unknown | 2,000 |  |  | 0 |
|  | Peak flow disposable mouth pcs | 1d | Unknown | 5 |  |  | 0 |
|  | Glucometer, with displayed unit "mg/dL", with 3 batteries (3V, type CR 2032) | 1e | 3 | 11 |  |  | 0 |
|  | Peak flow meter | 1e | 2 | 2 |  |  | 0 |
|  | Stethoscope complete binaural. | 1e | 5 | 4 |  |  | 0 |
|  | Upper arm blood pressure sphygmomanometer for adult (aneroid) with 1 spare cuff for adult in the package. | 1e | 3 | 9 |  |  | 0 |
|  | Diagnostic set, combined Otoscope & Opthalmoscope | 1e | 3 | 3 | Available AND Functional | Unknown | Unknown |
|  | Body tape measure (Figure Finder tape) | 1e | 3 | 3 |  |  |  |
|  | Pen flashlight prefocus or portable torch lamp | 1e | 3 | 3 |  |  |  |
|  | Thermometer clinical, armpit or pen typedigital, 32-43ºC | 1e | 5 | 5 |  |  |  |
| Malakal PHCC | Insulin syringes, 3 pieces, IU-100 | 1b | Not ordered | 3,200 | - |  | 0 |
|  | Urine test strips for ketones, glucose and protein | 1c | Unknown | 500 | Available AND Functional |  | 0 |
|  | Blood Glucose strips | 1d |  | 1,000 |  |  | 0 |
|  | Lancet, blood, ster, disp, | 1d |  | 3,000 |  |  | 0 |
|  | Peak flow disposable mouth pcs | 1d |  | 5 |  |  | 0 |
|  | Glucometer, with displayed unit "mg/dL", with 3 batteries (3V, type CR 2032) | 1e | 3 | 10 |  |  | 0 |
|  | Peak flow meter | 1e | 2 | 2 |  |  | 0 |
|  | Stethoscope complete binaural. | 1e | 5 | 5 |  |  | 0 |
|  | Upper arm blood pressure sphygmomanometer for adult (aneroid) with 1 spare cuff for adult in the package. | 1e | 3 | 3 |  |  | 0 |
|  | Diagnostic set, combined Otoscope & Ophthalmoscope | 1e | 3 | 3 |  |  | 0 |
|  | Body tape measure (Figure Finder tape) | 1e | 3 | 3 |  |  | 0 |
|  | Pen flashlight prefocus or portable torch lamp | 1e | 3 | 3 |  |  | 0 |
|  | Thermometer clinical, armpit or pen type digital, 32-43ºC | 1e | 5 | 5 |  |  | 0 |

*Average Monthly Consumption

**Out of stock (OOS)
