# Supplementary material for: Assessment of the WHO non-communicable diseases kit for humanitarian emergencies in South Sudan: a retrospective, prospective, observational study
Source: Confl Health. 2023 Jun 5;17:27. doi: 10.1186/s13031-023-00525-w (PMC10241119; doi:10.1186/s13031-023-00525-w)
Supplement: Supplementary file 3 — Supplementary Material 3 [file 13031_2023_525_MOESM3_ESM.docx]

**Supplementary Material 3: Demographics and Key findings of the Healthcare Workers Survey**

A total of 40 participants completed both baseline and endline surveys. eighty six percent of baseline respondents were females, while only 10% of endline respondents were males. Figures 1 and 2 illustrates the distribution of studied sample across all cadres during baseline and endline assessments. Most participants were Nurses (35%, n= 14), followed by clinical officers and laboratory technicians which both accounted for (18%, n=7). For physicians and health/medical assistants, each category weighted 8% (n=3) followed by the two pharmacists (5%). The “others” category found during endline was composed of three nursing assistants and one pharmacy assistant. Nearly 60% of the studied cohort reported having between three to five gender, cadres, years of experience as shown in figure 3. (Additional details are shown in Tables 1,2, and 3).

| Table 1. Respondents Cadres | | | | | |
| --- | --- | --- | --- | --- | --- |
| Cadre | *Baseline* | | | | |
|  | **Malakal PHCC** | **Munuki PHCC** | **Bentiu Hospital** | **Kotor PHCC** | ***Grand-Total*** |
| Physician/Doctor | 2 | - | - | - | ***2*** |
| Clinical Officer | - | - | - | 1 | ***1*** |
| Health/Medical Assistant | 1 | - | - | 1 | ***2*** |
| Lab technician | 1 | 1 | 1 | 1 | ***4*** |
| Nurse | - | 1 | 1 | 3 | ***5*** |
| Pharmacist | - | 1 | - | - | ***1*** |
| *Sub-Total* | **4** | **3** | **2** | **6** | **15** |
| *Endline* | | | | | |
| Cadre | **Malakal PHCC** | **Bentiu Hospital** | **Juba Teaching Hospital** | | ***Grand-Total*** |
| Physician/Doctor | - | - | 1 | | ***1*** |
| Nurse | 2 | 5 | 2 | | ***9*** |
| Pharmacist | - | 1 | - | | ***1*** |
| Clinical Officer | 2 | 3 | 1 | | ***6*** |
| Health/Medical Assistant | 1 | - | 1 | | ***2*** |
| Lab technician | - | 2 | - | | ***2*** |
| Other | - | 4 | - | | ***4*** |
| *Sub Total* | **5** | **15** | **5** | | **25** |

| Table 2. Respondents Genders | | |
| --- | --- | --- |
|  | Baseline | Endline |
| Males | 2 | 18 |
| Females | 13 | 7 |
| Total | **15** | **25** |

| Table 3. Key Findings of The Provider Survey in South Sudan | | |
| --- | --- | --- |
| Key Finding | Baseline (n=15) | Endline(n=25) |
| Staff clinical experience | Nearly 60% of the studied cohort reported having between three to five years of experience | |
| No previous NCD training - all staff | 67% | 68% |
| No previous NCD training  (Prescribers only) ^A,B^ | 100% | 40% |
| Average scores for basic knowledge questions (all staff) | 78% | NA |
| Average scores for NCDK contents questions (Prescribers only) ^A,B^ | 28% | 37% |
| Average scores for Clinical questions (Prescribers only) ^B^ | 27% | 66% |
| Average scores for NCDK contents questions (Non-prescribers ) ^C^ | NA | 21% |
| Average scores for Clinical questions (Non-prescribers) ^C^ | NA | 56% |
| A) Number of participating prescribers at baseline were only two; both from the same facility.  B) Number of participating prescribers at endline were (n=10); from all facilities.  C) Number of participating non-prescribers at endline were (n=15); from all facilities. | | |

Overall, the average percentage of basic knowledge questions section answered correctly among all staff in the baseline assessment (n=15) was 78%. The most frequently incorrectly answered questions were around the danger signs for complications of diabetes and which conditions may be prevented through eating a balanced diet (many incorrectly selected HIV & Malaria). The basic knowledge section was not included in the endline version of the survey. The average scores of the two prescribing providers (a physician and a clinical officer) who responded at baseline to the NCDK content and clinical questions were 28% and 27% for both sections, respectively. The other clinical officer responded “no” to the question asking if the respondent prescribes medicines to patients, and thus was not prompted to answer the NCDK content/clinical questions. At endline, there were five nurses, four clinical officers and one physician who reported prescribing NCD medicines; they (n=10) scored 39% on average in the NCDK content, and 68% in the clinical questions. Non-prescribers (n=15) had corresponding scores of 24% on NCDK content and 56% on NCD clinical questions. Both clinical officers incorrectly answered most questions related to the NCDK contents included at baseline regarding the use of Beclomethasone inhalers, epilepsy treatments (Sodium Valproate and Carbamazepine), Metformin, Levothyroxine, Glibenclimide, Risperidone, Aspirin, and Beta-blocker drugs such as Bisoprolol. Further, both prescribing staff skipped questions in the clinical practice section regarding the point at which emergency treatment should be started after onset of a seizure, treatment for myocardial infarction, common complications of diabetes, treatment for diabetic ketoacidosis, and pre-eclampsia diagnosis. (Additional details can be found in figures 4 and 5).

At endline, all prescribing participants skipped some of the clinical questions. These questions included the ones related to prescribing/stock, questions about supplies, and the questions regarding hypothetical treatments or investigative steps for cases of suspected stroke, diabetic ketoacidosis, organ damage, and pre-eclampsia. However, for the NCDK content section, respondents more often correctly answered questions related to medications not used to treat hypertension such as Glibenclamide or Glyburide, and which patients should not receive non-steroidal anti-inflammatory drugs (NSAIDs). They incorrectly answered other questions related to the use of Metformin or beta-blocker drugs such as Bisoprolol, and the recommended starting dosage of Risperidone for acute psychosis. In the NCD clinical knowledge portion, respondents correctly answered questions related to the causes or risk factors of hypertension, symptoms of diabetes or respiratory disease, and others related to the prevention of diabetes, COPD, and hypertension. Importantly, respondents incorrectly answered most questions related to treatments and diagnoses, such as management of COPD exacerbation versus asthma or drugs that are not recommended for patients who have had a myocardial infarction.
